# Fabrication of Ag and Mn Co-Doped Cu_2_ZnSnS_4_ Thin Film

**DOI:** 10.3390/nano9111520

**Published:** 2019-10-25

**Authors:** Lei Qiu, Jiaxiong Xu, Xiao Tian

**Affiliations:** School of Materials and Energy, Guangdong University of Technology, Guangzhou 510006, China; 2111602071@mail2.gdut.edu.cn (L.Q.); 2111802108@mail2.gdut.edu.cn (X.T.)

**Keywords:** Cu_2_ZnSnS_4_, Ag and Mn co-doping, thin films, sol–gel preparation

## Abstract

Ag and Mn dopants were incorporated into Cu_2_ZnSnS_4_ thin film to reduce defects in thin film and improve thin film properties. Sol–gel and spin-coating techniques were employed to deposit Ag and Mn co-doped Cu_2_ZnSnS_4_ thin films. The structures, compositions, morphologies, and optical properties of the co-doped thin films were characterized. The experimental results indicate the formation of kesterite structure without Ag and Mn secondary phases. The amount of Ag in the thin films is close to that in the sols. The co-doped Cu_2_ZnSnS_4_ thin films have an absorption coefficient of larger than 1.3 × 10^4^ cm^−1^, a direct optical band gap of 1.54–2.14 eV, and enhanced photoluminescence. The nonradiative recombination in Cu_2_ZnSnS_4_ thin film is reduced by Ag and Mn co-doping. The experimental results show that Ag and Mn incorporation can improve the properties of Cu_2_ZnSnS_4_ thin film.

## 1. Introduction

In recent years, the quaternary semiconducting Cu_2_ZnSnS_4_ has been recognized as a candidate to replace Cu(In,Ga)Se_2_ absorber due to its promising optical and electronic properties and its earth-abundant and non-toxic component elements [1,2]. The absorption coefficient of Cu_2_ZnSnS_4_ in the visible region is larger than 1 × 10^4^ cm^−1^. The direct band gap of Cu_2_ZnSnS_4_ is around 1.50 eV, which matches with the suitable value for absorber application in solar cells [3,4].

The record conversion efficiencies of Cu_2_ZnSnS_4_ and Cu_2_ZnSn(S,Se)_4_ thin-film solar cells are 11.0% [5] and 12.6% [6], respectively, which are still lower than that of competitive Cu(In,Ga)Se_2_ thin-film solar cell. Further improvement is necessary for Cu_2_ZnSnS_4_-based thin-film solar cell. The low efficiency of Cu_2_ZnSnS_4_ thin-film solar cell is attributed to the poor crystallinity of Cu_2_ZnSnS_4_, easy formation of secondary phases in Cu_2_ZnSnS_4_, defect states in Cu_2_ZnSnS_4_ and heterojunction interface, unfavorable band alignment at the buffer/absorber and absorber/back electrode interfaces, etc. [7].

Cation substitution is a useful method to enhance the properties of Cu_2_ZnSnS_4_ thin film and solar cell. The Cu^+^, Zn^2+^, and Sn^4+^ ions in Cu_2_ZnSnS_4_ can be substituted by extrinsic ions. Ag doping can occupy the Cu site in the Cu_2_ZnSnS_4_ lattice to reduce the harmful Cu_Zn_ anti-site and V_Cu_ defects because the formation energy of Ag_Zn_ defect is higher than that of Cu_Zn_ defect [8,9,10,11]. In addition, the band gap of Cu_2_ZnSnS_4_ can be adjusted by Ag doping [12,13].

The full or partial substitution of Zn by Cd, Fe, Mn, Mg, or Co has also been reported to increase the absorption of Cu_2_ZnSnS_4_, reduce the Cu_Zn_ anti-site defect and ZnS secondary phase, and enhance the crystallinity of Cu_2_ZnSnS_4_ and conversion efficiency of Cu_2_ZnSnS_4_ thin-film solar cell [14,15,16,17,18]. The efficiency of Cd-doped Cu_2_ZnSnS_4_ solar cell has exceeded 11% [16].

Up to now, most of reports about cation substitution of Cu_2_ZnSnS_4_ have utilized single doping, that is, only one dopant is used for substitution. Although the single substitution has demonstrated beneficial effects on Cu_2_ZnSnS_4_, there are still some issues. Ag substitution increases the band gap of Cu_2_ZnSnS_4_, leading to reduced wavelength of the absorption edge of Cu_2_ZnSnS_4_. The effect of reduced defects by Zn site substitution is limited. The optimum substitution ratio of single doping is in a narrow range. It needs precise composition regulation during thin film preparation. Compared with single doping, if different cations dope in Cu_2_ZnSnS_4_ to substitute different sites with different mechanisms, this is expected to further improve the properties of Cu_2_ZnSnS_4_ thin film. To the best of our knowledge, there is only one report about Ag and Cd co-doped Cu_2_ZnSnS_4_ [19]. However, Cd is a toxic element.

In this work, Cu_2_ZnSnS_4_ thin films were co-doped by Ag and Mn dopants using sol–gel and spin-coating methods. The Ag and Mn atoms substitute the Cu and Zn sites, respectively. Ag is commonly used for Cu substitution in Cu_2_ZnSnS_4_. Mn is earth-abundant and non-toxic. The sol–gel process has an advantage in thin film doping because the chemical homogeneity of sol can reach molecular or even atomic level. The structural, compositional, morphological, and optical properties of Ag and Mn co-doped Cu_2_ZnSnS_4_ thin films were characterized.

## 2. Materials and Methods

**Figure 1:**
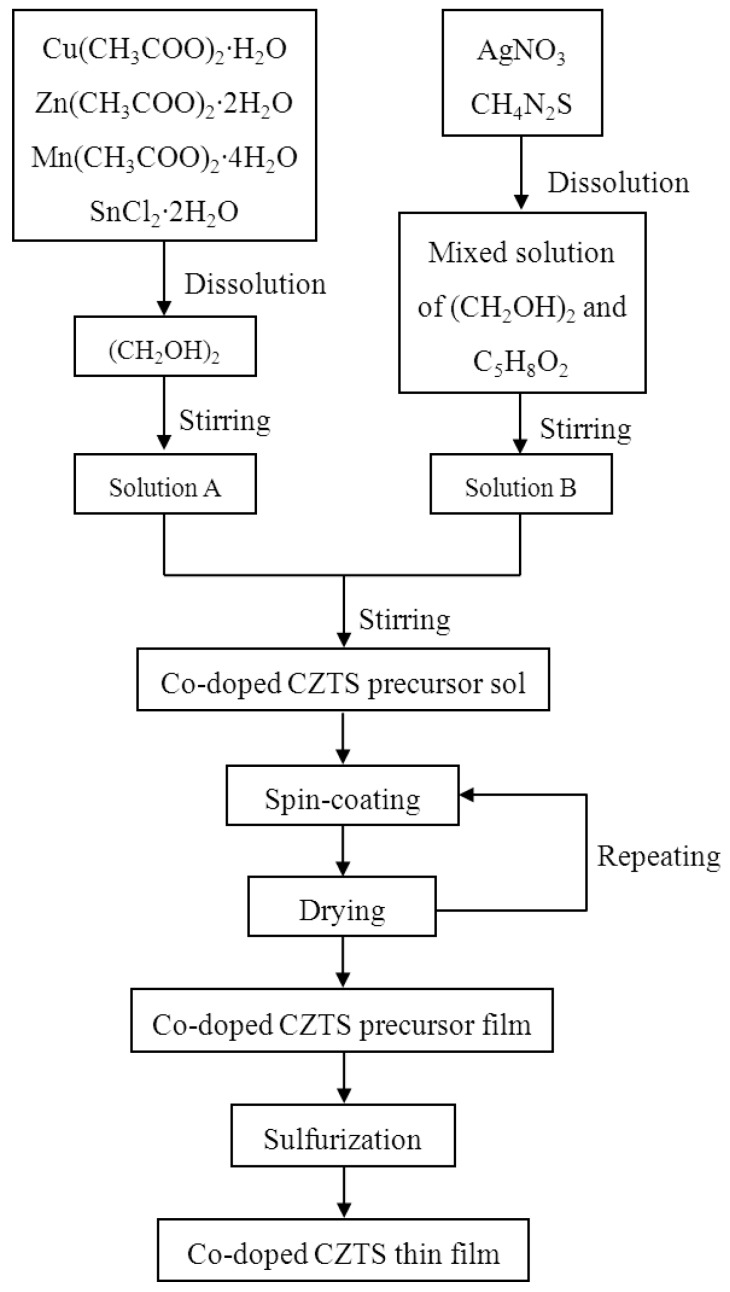
Flow chart for the fabrication process of Ag and Mn co-doped Cu_2_ZnSnS_4_ thin films.

Figure 1 illustrates the fabrication process of Ag and Mn co-doped Cu_2_ZnSnS_4_ thin films. Cu(CH_3_COO)_2_·H_2_O, Zn(CH_3_COO)_2_·2H_2_O, SnCl_2_·2H_2_O, CH_4_N_2_S, and C_5_H_8_O_2_ were purchased from Shanghai Aladdin Bio-Chem Technology Co., LTD, Shanghai, China; Mn(CH_3_COO)_2_·4H_2_O, (CH_2_OH)_2_, and AgNO_3_ were purchased from Guangzhou Chemical Reagent Factory, Guangzhou, China, Sinopharm Chemical Reagent Co., Ltd., Shanghai, China, and Guangdong Guanghua Sci-Tech Co., Ltd., Shantou, China, respectively. Cu(CH_3_COO)_2_·H_2_O, Zn(CH_3_COO)_2_·2H_2_O, Mn(CH_3_COO)_2_·4H_2_O, and SnCl_2_·2H_2_O were sequentially dissolved in an organic (CH_2_OH)_2_ solvent, and then stirred at 60 °C for 2 h to obtain a milky solution, which was labeled as solution A. AgNO_3_ and CH_4_N_2_S were added into a mixed solution of (CH_2_OH)_2_ and C_5_H_8_O_2_ (the volume ratio of (CH_2_OH)_2_ to C_5_H_8_O_2_ was 5:1). After stirring for 2 h at 60 °C, a homogeneous yellow solution labeled as solution B was formed. Then, solution A was added into solution B and stirred at 60 °C for 15 min to obtain a homogeneous precursor sol. The sol samples with different Ag amounts were prepared with atomic ratios of Ag/(Ag + Cu) = 0, 1/3, 2/3, and 1, Mn/(Mn + Zn) = 1/3, (Ag + Cu)/(Mn + Zn + Sn) = 0.85, (Mn + Zn)/Sn = 1.15, and S/(Ag + Cu) = 4. The prepared sols were aged for 96 h at atmospheric pressure without heating. After that, the sols were spin-coated onto FTO (fluorine-doped tin oxide)-coated glass substrates to fabricate Cu_2_ZnSnS_4_ precursor films. The spin-coating was performed at a rotation speed of 800 rpm for 20 s and then 3500 rpm for 35 s. The films were then dried on a hot plate at 250 °C for 4 min to remove residual organics. The coating and drying were repeated 10 times. Finally, the Cu_2_ZnSnS_4_ precursor films were sulfurized to obtain the final co-doped Cu_2_ZnSnS_4_ thin films. The precursor films and 1 g sulfur powders were putted in a quartz boat which was placed in the center of a tube furnace. The sulfurization was performed in a N_2_ + S atmosphere; the sulfurization temperature and sulfurization time were 530 °C and 60 min, respectively.

The structures of prepared thin films were measured by X-ray diffractometer (XRD, D/MAX-Ultima IV, Rigaku, Tokyo, Japan) using Cu-kα radiation with a wavelength of 0.154 nm). A Raman scattering spectrometer (FEX, NOST Company Limited, Seongnam, Korea) was used for better detection of phase structure in the thin film. The excitation wavelength of Raman measurement was 532 nm. A field emission scanning electron microscope (FESEM, SU8010, Hitachi, Tokyo, Japan) was used to observe the surface and cross-section morphologies of the co-doped Cu_2_ZnSnS_4_ thin films. The atomic ratios of thin films were measured by energy dispersive spectroscopy (EDS, SDD3030, IXRF Systems, Austin, TX, USA) which was attached to FESEM. The reflectance and transmittance of thin films were identified by a UV-Vis spectrophotometer (UV-3600 Plus, Shimadzu, Kyoto, Japan and TU1810, Pgeneral, Beijing, China). The photoluminescence (PL) properties of Cu_2_ZnSnS_4_ were measured by a fluorescence spectrophotometer (Fluorolog-3, HORIBA Instruments Incorporate, Irvine, CA, USA).

## 3. Results and Discussion

**Figure 2:**
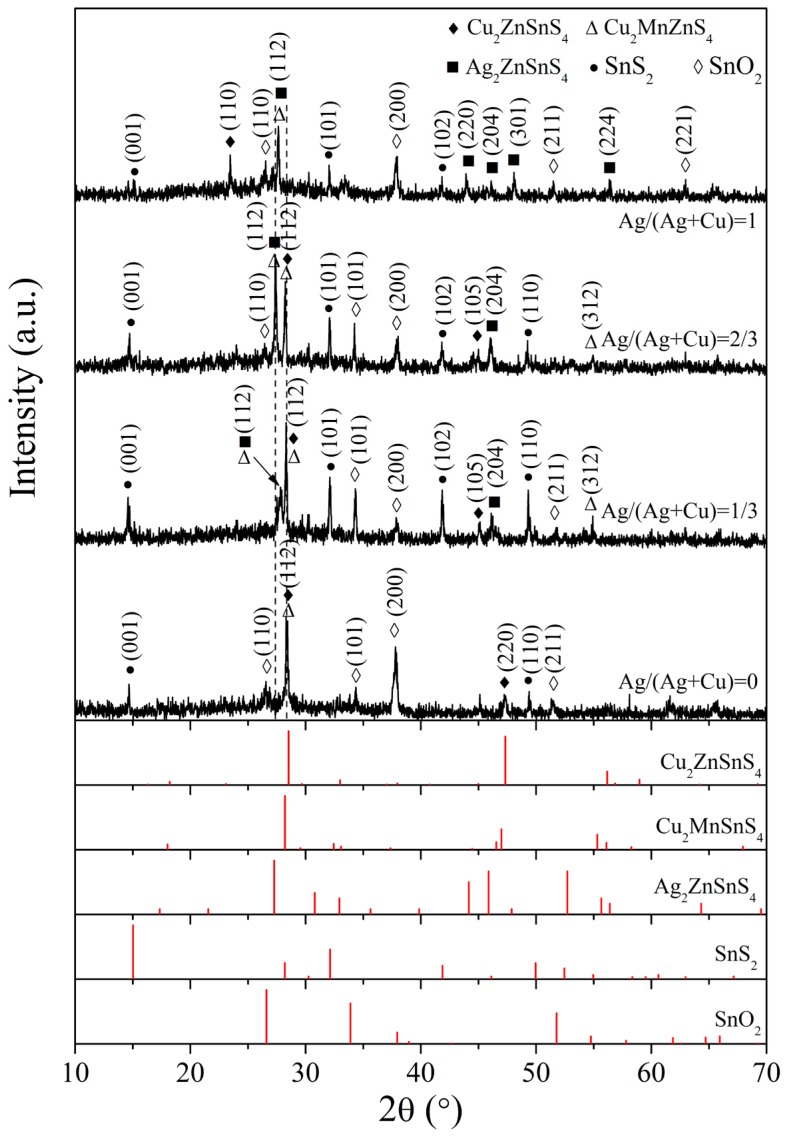
XRD patterns of Ag and Mn co-doped Cu_2_ZnSnS_4_ thin films with different Ag/(Ag + Cu) ratios and the standard XRD peaks for Cu_2_ZnSnS_4_, Cu_2_MnSnS_4_, Ag_2_ZnSnS_4_, SnS_2_, and SnO_2_.

Figure 2 shows the XRD patterns of Ag and Mn co-doped Cu_2_ZnSnS_4_ thin films with different Ag amounts and the standard XRD peaks for Cu_2_ZnSnS_4_, Cu_2_MnSnS_4_, Ag_2_ZnSnS_4_, SnS_2_, and SnO_2_. The XRD pattern of Mn-doped Cu_2_ZnSnS_4_ thin film without Ag shows the strongest peak at 28.3°, which is between the positions of the peaks attributed to the (112) plane of Cu_2_MnSnS_4_ (28.2°) and the (112) plane of Cu_2_ZnSnS_4_ (28.5°). The diffraction peak at 47.2° in the pattern is attributed to the (220) plane of Cu_2_ZnSnS_4_. These results indicate the incorporation of Mn into Cu_2_ZnSnS_4_ lattice and the preferred orientation along the (112) plane. The peaks belonging to the secondary phase of SnS_2_ are detected at 14.7° and 49.4°. The SnO_2_ peaks originate from the FTO substrate. When Ag is introduced into Mn-doped Cu_2_ZnSnS_4_ with Ag/(Ag + Cu) = 1/3, the dominant diffraction peak is still located at 28.3° and becomes stronger. In addition, a weak peak appears at 27.8°, which is between the standard (112) peak of Ag_2_ZnSnS_4_ (27.3°) and the (112) peak of Cu_2_MnSnS_4_ (28.2°). Therefore, Ag and Mn co-doped Cu_2_ZnSnS_4_ structure is formed. When the atomic ratio of Ag/(Ag + Cu) increases to 2/3, the two peaks at around 28° slightly shift to the low diffraction angle direction due to the increase in the amount of Ag. The left side peak at 27.4° is stronger than that at 28.2° because the Ag amount is greater than the Mn amount in this sample. For the thin film with full substitution of Ag, the peak at 28.2° disappears. A main but weak peak located at 27.6° is related to Ag_2_ZnSnS_4_ or Cu_2_MnSnS_4_ phases. All the co-doped Cu_2_ZnSnS_4_ thin films have preferred orientation along the (112) plane. Secondary phase of SnS_2_ can be detected in all thin films.

**Figure 3:**
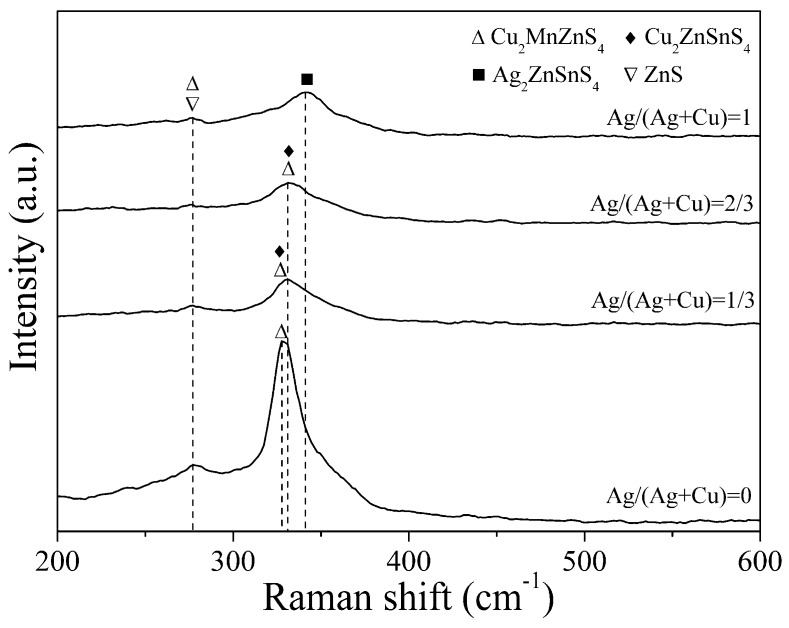
Raman spectra of Ag and Mn co-doped Cu_2_ZnSnS_4_ thin films with different Ag/(Ag + Cu) ratios.

Figure 3 shows the Raman spectra of Ag and Mn co-doped Cu_2_ZnSnS_4_ thin films with different Ag amounts. The strongest Raman characteristic peak of Mn single-doped Cu_2_ZnSnS_4_ thin film is located at 327 cm^−1^, matching with the reported position of the Cu_2_MnZnS_4_ peak [20,21]. The thin films with Ag/(Ag + Cu) = 1/3 and Ag/(Ag + Cu) = 2/3 show a Cu_2_ZnSnS_4_ or Cu_2_MnZnS_4_ peak at 331 cm^−1^ [20,22,23]. For the thin film with full Ag substitution, the Raman peak at 342 cm^−1^ is near the reported Ag_2_ZnSnS_4_ peak [24]. The weak peaks at 277 cm^−1^ in all thin films originate from the vibrational mode of Cu_2_MnZnS_4_ [23] or ZnS [25]. The strongest Raman peak shifts to a higher Raman shift direction with increasing Ag amount due to the change of phase structure from Cu_2_MnZnS_4_ to Ag_2_ZnSnS_4_. The SnS_2_ secondary phase detected by XRD has Raman shifts of 215 cm^−1^ and 315 cm^−1^ [26]. However, all the prepared thin films do not show the Raman peak of SnS_2_. Since Raman scattering measurement is surface-sensitive, the SnS_2_ phase may locate near the back of thin film and cannot be detected by Raman measurement. Ag or Mn secondary phase is absent in the Raman spectra. The measured results of XRD and Raman confirm the successful formation of Ag and Mn co-doped kesterite Cu_2_ZnSnS_4_ thin films without a secondary phase related to Ag or Mn.

Table 1 provides the atomic percentages of Ag, Cu, Mn, Zn, Sn, and S in the prepared samples. The calculated atomic ratios of Ag/(Ag + Cu), Mn/(Mn + Zn), (Cu + Ag)/(Mn + Zn), and S/(Ag + Cu) of each sample are also given in Table 1. During EDS measurement, only the Ag, Cu, Mn, Zn, Sn, and S elements in the thin film were considered and the Si, O, and other elements were ignored. It is noted that the atomic percentage of Sn in the thin film is lower than the measured value because the Sn element in FTO contributes to the measured result. The Ag/(Ag + Cu) ratios of Cu_2_ZnSnS_4_ thin films are close to those of sols, indicating the effective incorporation of Ag into Cu_2_ZnSnS_4_ thin films. However, the measured Mn/(Mn + Zn) ratios of thin films are lower than those of sols due to the precipitation of Mn during sol preparations. The value of S/(Ag + Cu) approaches the stoichiometry of 2 with increasing Ag amount.

**Figure 4:**
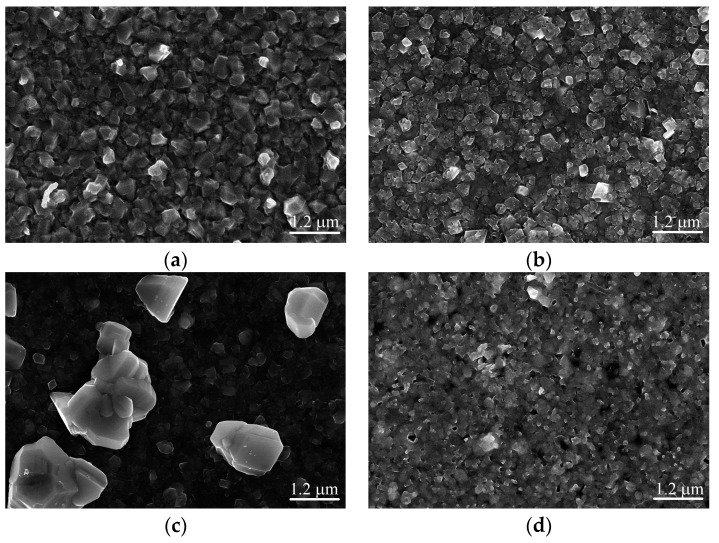
Surface SEM images of Ag and Mn co-doped Cu_2_ZnSnS_4_ thin films with (**a**) Ag/(Ag + Cu) = 0; (**b**) Ag/(Ag + Cu) = 1/3; (**c**) Ag/(Ag + Cu) = 2/3; and (**d**) Ag/(Ag + Cu) = 1.

**Figure 5:**
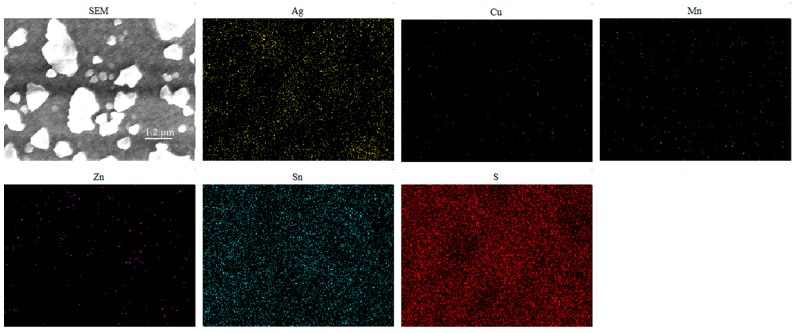
EDS mapping results for Ag and Mn co-doped Cu_2_ZnSnS_4_ thin film with Ag/(Ag + Cu) = 2/3.

Figure 4 shows the surface SEM images of Ag and Mn co-doped Cu_2_ZnSnS_4_ thin films with different Ag amounts. In Figure 4a, for the Mn-doped Cu_2_ZnSnS_4_ thin film without Ag doping, grains with size of about 300 nm distribute on the thin film surface. After Ag incorporation with Ag/(Ag + Cu) = 1/3, the thin film surface becomes more compact and the grain size is about 280 nm. When Ag/(Ag + Cu) = 2/3, the surface of thin film is crack-free and consists of isolated, discontinuous, and large grains with a maximum size of around 2 μm and small grains with size of about 300 nm. EDS mapping was further performed for this sample and the results are given in Figure 5. Although there is slight inhomogeneity of element distribution in the thin film, all the six elements distribute in both large and small grains. Therefore, both grains are Ag and Mn co-doped Cu_2_ZnSnS_4_. In Figure 4d, when Cu is fully substituted by Ag the grain size significantly reduces and the grains become fuzzy. Meanwhile, pinholes increase and enlarge in the thin film surface. As revealed from XRD results, the co-doped thin films contain a secondary phase of SnS_2_. Since SnS_2_ is volatile at high temperature, these pinholes may result from the evaporation of SnS_2_ during thin film fabrications. There is residual SnS_2_ in the final thin film.

**Figure 6:**
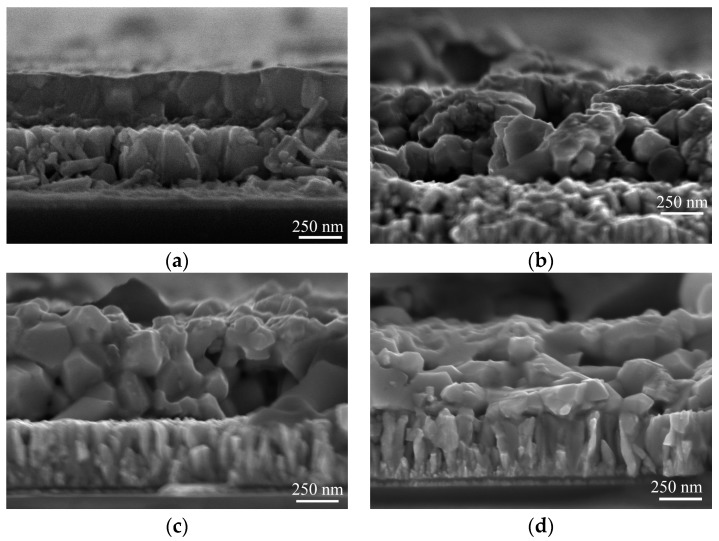
Cross-section SEM images of Ag and Mn co-doped Cu_2_ZnSnS_4_ samples with (**a**) Ag/(Ag + Cu) = 0; (**b**) Ag/(Ag + Cu) = 1/3; (**c**) Ag/(Ag + Cu) = 2/3; and (**d**) Ag/(Ag + Cu) = 1.

The cross-section SEM images of Cu_2_ZnSnS_4_ samples in Figure 6 show Cu_2_ZnSnS_4_/FTO/glass stacked structure. The thin film with Mn single doping has homogeneous, smooth, and compact cross-section morphology, but the grain is hard to detect. After Ag co-doping, grainy morphologies can be seen in the cross-sections of thin films. The thickness of co-doped Cu_2_ZnSnS_4_ thin films is 650 nm. There are some pinholes in the co-doped thin films and at the Cu_2_ZnSnS_4_/FTO interfaces, which may result from the evaporation of SnS_2_ secondary phase as mentioned previously.

**Figure 7:**
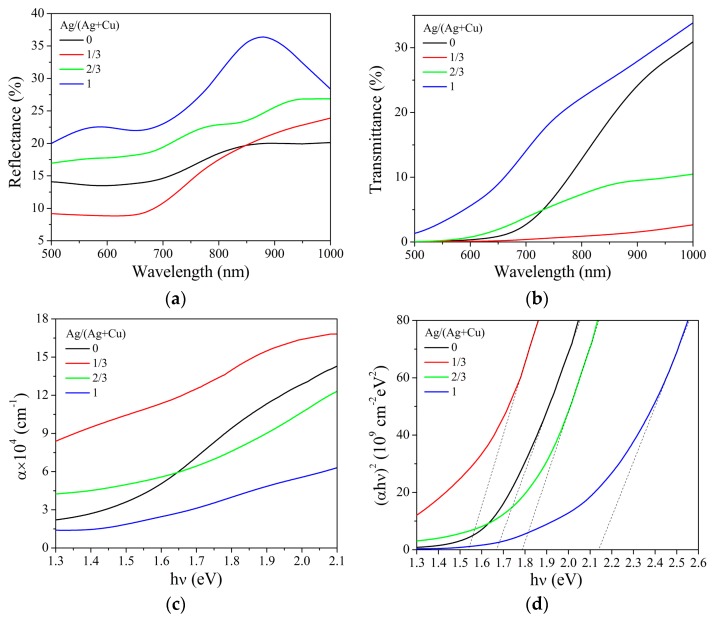
The (**a**) reflectance; (**b**) transmittance; (**c**) absorption coefficient; and (**d**) (*αhυ*)^2^ versus *hυ* relations of Ag and Mn co-doped Cu_2_ZnSnS_4_ thin films with different Ag/(Ag + Cu) ratios.

Figure 7 shows the reflectance (*R*), transmittance (*T*), absorption coefficient (*α*), and (*αhυ*)^2^ versus *hυ* relations of prepared thin films, where *hυ* is photon energy. The reflectance of all Cu_2_ZnSnS_4_ samples in the wavelength range of 500 to 1000 nm is lower than 37%. The average reflectances of Cu_2_ZnSnS_4_ thin films with Ag/(Ag + Cu) ratios of 0, 1/3, 2/3, and 1 are 16.7%, 15.0%, 21.5%, and 27.5%, respectively. The transmittance of samples decreases with the reduction of wavelength due to the absorption in Cu_2_ZnSnS_4_. The absorption coefficient of thin film is calculated by the following equation [27]:

(1) $$\alpha =-\frac{1}{d}\ln\left(\frac{T^{2}-{\left(1-R\right)}^{2}+\sqrt{4T^{2}+{\left({\left(1-R\right)}^{2}-T^{2}\right)}^{2}}}{2T}\right),$$

where *d* is the thickness of thin film. As shown in Figure 7c, the absorption coefficients of Ag and Mn co-doped Cu_2_ZnSnS_4_ thin films are larger than 1.3 × 10^4^ cm^−1^ for photon energy greater than 1.3 eV. Compared with Mn single-doped thin film, Ag co-doping with a suitable amount can increase the absorption coefficient of Cu_2_ZnSnS_4_ thin films. According to the relation of (*αhυ*)^2^ = C(*hυ* – *E_g_*), where C is a constant and *E_g_* is direct optical band gap, the *E_g_* of Cu_2_ZnSnS_4_ thin film can be obtained from the (*αhυ*)^2^–*hυ* curves in Figure 7d. The intercept on the *hυ* axis of the extrapolation of the linear region of (*αhυ*)^2^–*hυ* curve gives the *E_g_* values of 1.67, 1.54, 1.78, and 2.14 eV for Cu_2_ZnSnS_4_ thin films with Ag/(Ag + Cu) ratios of 0, 1/3, 2/3, and 1, respectively. The *E_g_* of co-doped Cu_2_ZnSnS_4_ thin film increases with the increasing Ag/(Ag + Cu) ratio due to Ag doping. It is reported that the band gap of Ag single-doped Cu_2_ZnSnS_4_ thin film becomes increasingly wide with the increase of Ag concentration [12,13].

**Figure 8:**
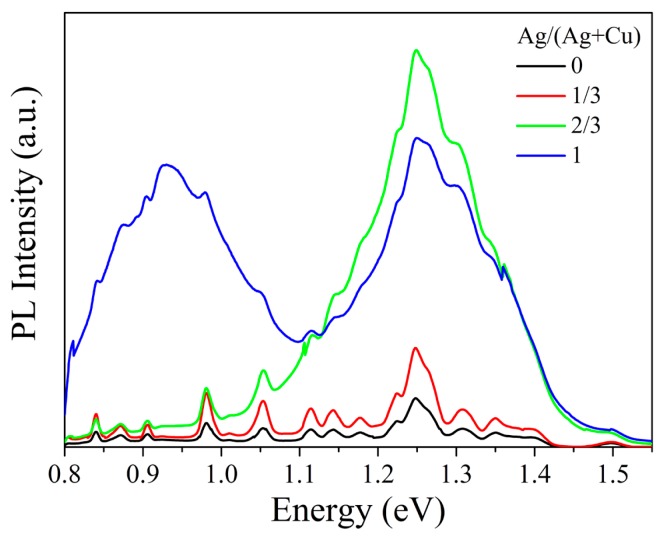
Photoluminescence spectra of Mn single-doped and Ag and Mn co-doped Cu_2_ZnSnS_4_ thin films with different Ag/(Ag + Cu) ratios.

Figure 8 shows photoluminescence spectra of single-doped and co-doped Cu_2_ZnSnS_4_ thin films. Compared with the Mn single-doped Cu_2_ZnSnS_4_ thin film, all the Ag and Mn co-doped Cu_2_ZnSnS_4_ thin films show enhanced photoluminescence, especially the thin films with Ag/(Ag + Cu) ratios of 2/3 and 1. This indicates that Ag and Mn co-doping can reduce the harmful defect states and nonradiative recombination in Cu_2_ZnSnS_4_ thin film by double cation substitutions.

Compared with the Mn single-doped Cu_2_ZnSnS_4_ thin films in this study and previously reported results [28,29,30], a further incorporation of Ag with suitable doping concentration can promote grain growth, increase the absorption coefficient of thin film, expand the adjustment range of the band gap of thin film, and reduce the nonradiative recombination in the thin film.

## 4. Conclusions

Ag and Mn co-doped Cu_2_ZnSnS_4_ thin films with different Ag amounts were successfully fabricated on FTO-coated glass substrates by sol–gel and spin-coating techniques. The XRD and Raman measurements reveal the formation of Ag and Mn co-doped Cu_2_ZnSnS_4_ with preferred orientation of the (112) plane and without Ag or Mn secondary phase. The atomic ratios of Ag/(Ag + Cu) in the Cu_2_ZnSnS_4_ thin films are close to those of original sols. Ag co-doping can enhance the grain size and absorption coefficient of Cu_2_ZnSnS_4_ thin films. The thickness and direct optical band gap of Ag and Mn co-doped Cu_2_ZnSnS_4_ thin films are 650 nm and 1.54–2.14 eV, respectively. The Ag and Mn co-doped Cu_2_ZnSnS_4_ thin films show an enhanced photoluminescence property, indicating the beneficial effects of reduced defects and nonradiative recombination by Ag and Mn co-doping. This work reveals potential advantages of Ag and Mn co-doping on Cu_2_ZnSnS_4_ thin film.

## Figures and Tables

**Table 1:** The compositions of Ag and Mn co-doped Cu_2_ZnSnS_4_ thin films.

Composition	Ag/(Ag + Cu) in Sol			
	0	1/3	2/3	1
Ag (at.%)	0	5.88 ± 0.03	11.93 ± 0.44	21.09 ± 0.82
Cu (at.%)	17.46 ± 0.13	9.37 ± 0.15	6.07 ± 0.44	0.33 ± 0.03
Mn (at.%)	2.29 ± 0.15	2.55 ± 0.07	2.37 ± 0.10	2.04 ± 0.16
Zn (at.%)	7.06 ± 0.21	6.07 ± 0.45	5.58 ± 0.61	5.71 ± 0.30
Sn (at.%)	25.89 ± 0.30	29.65 ± 0.68	26.29 ± 0.87	21.90 ± 0.41
S (at.%)	47.31 ± 0.52	46.50 ± 0.44	47.76 ± 1.04	48.93 ± 1.44
Ag/(Ag + Cu)	0	0.39	0.66	0.98
Mn/(Mn + Zn)	0.24	0.30	0.30	0.26
(Cu + Ag)/(Mn + Zn)	1.87	1.77	2.26	2.76
S/(Ag + Cu)	2.71	3.05	2.65	2.28
